# Remarks on Dr. Bateman's Description of Elephantiasis

**Published:** 1815-02

**Authors:** 


*For the Medical and Physical Journal.*

### *Remarks on Dr. Bateman's Description of Elephantiasis.*

AS you have invited communications on the subject of Elephantiasis, and as, with other students, it is my wish to make the most of my time whilst in London, I shall be thankful for a small corner in your Journal.

On examining the last edition of Dr. Bateman, I observe that he now tells his readers he has seen two instances of the disease. In his former edition, he acknowledges that he had seen none. At that time, therefore, we were prepared to meet with only the descriptions of other writers; but, after the author had seen two cases, we might have expected his own account. Instead of which, even the point which he formerly considered doubtful, but on which he has now had an opportunity of satisfying himself, is left in the same uncertainty as before. "Dr. Adams," he still says, "contrary to all other authorities, observed, in the lazars of Madeira, an actual wasting of the generative organs in men who had been seized subsequent to the age of puberty, and a want of the usual evolution of them in those who had been attacked previous to that period." "Is the Elephantiasis of Madeira," he adds, "now less virulent than that of former times? has it undergone some change in its character? or is the ancient account of the disease incorrect?"*

Either these inquiries are important, or they should have been omitted. If they are important, why have we not Dr. Bateman's answer to them as far as his knowledge extends? That he has now seen the disease, we have his own confession. That he has seen the case in this hospital, I can answer. Why then have we not a solution of this inquiry as far as that case informs him? Why are we still referred to contradictory accounts from authors whom Dr. Bateman

* Synopsis, 3d edit. p. 303.

 professes professes to undervalue, instead of his own description of what he has seen, or, which would have been the same, a transcript of Dr. Adams's description, or of the copy made by Dr. Bateman for the Cyclopœdia.

I will acknowledge to you the disgust I felt at still reading, that "accurate descriptions of Elephantiasis, &c. are still among the *desiderata* of the pathologist." So they must for ever remain, of this and of all other diseases, if we have only the hard names and contradictory accounts of Greeks, Moors, Arabians, and Africans, instead of a succinct description of what passed under the author's own eye.

As a young man, I may seem to be writing freely, but, recollecting how many of my brethren may be deprived of the present opportunity of seeing this extraordinary disease, I cannot conclude without assuring them that Dr. Adams's description is such as renders it impossible to mistake it; and that by that description alone, our house-surgeon, Mr. Ashburner, immediately recognised the complaint, when he saw the boy in the examining room. At your last account of him he was under the meazles. He has recovered and appears much better than before.

*A Student at St. Bartholomew's.*

---

